# Targeting Nuclear Receptors with Marine Natural Products

**DOI:** 10.3390/md12020601

**Published:** 2014-01-27

**Authors:** Chunyan Yang, Qianrong Li, Yong Li

**Affiliations:** State Key Laboratory of Cellular Stress Biology, Innovation Center of Cell Biology Research, School of Life Sciences, Xiamen University, Xiamen 361102, China; E-Mail: 21620121152327@stu.xmu.edu.cn

**Keywords:** nuclear receptors, marine natural products, ligands, screen, drug targets

## Abstract

Nuclear receptors (NRs) are important pharmaceutical targets because they are key regulators of many metabolic and inflammatory diseases, including diabetes, dyslipidemia, cirrhosis, and fibrosis. As ligands play a pivotal role in modulating nuclear receptor activity, the discovery of novel ligands for nuclear receptors represents an interesting and promising therapeutic approach. The search for novel NR agonists and antagonists with enhanced selectivities prompted the exploration of the extraordinary chemical diversity associated with natural products. Recent studies involving nuclear receptors have disclosed a number of natural products as nuclear receptor ligands, serving to re-emphasize the translational possibilities of natural products in drug discovery. In this review, the natural ligands of nuclear receptors will be described with an emphasis on their mechanisms of action and their therapeutic potentials, as well as on strategies to determine potential marine natural products as nuclear receptor modulators.

## 1. Introduction

Natural products, including compounds from plants, microbes, and marine species, have become major resources for bioactive agents and play a key role in the discovery of lead compounds for new drug research. The high hit rates in lead drug screening and large-scale structural diversity make marine natural products ideal candidates for drug discovery. However, these natural products are often in limited supply, and total synthesis remains difficult. Thus, the bottleneck is a shortage of raw material, which has made it very challenging for drug development from marine natural products. Driven by new developments in analytical technology, spectroscopy, and high-throughput screening, recent years have witnessed a renaissance in marine-based drug discovery since the first marine drug (Ziconotide) came out [1,2]. In addition to Ziconotide for the treatment of pain, Trabectedin is another successful marine drug for anticancer therapies [3,4].

One key approach in drug discovery is to identify a drug target associated with a particular disease and to screen for lead compounds that are able to appropriately regulate this target protein. A drug target is a functional region of a protein for which a significant fraction of family members have been successfully targeted by drugs. The most important feature of drug targets is that they are able to respond to small molecules including intracellular metabolites and xenobiotics, such as certain drugs. Rhodopsin-like GPCRs, certain ion-channel domains, and nuclear receptors (NRs) are the most successful molecular targets in the history of drug discovery [5]. Nuclear receptors, consisting of 48 members in humans, are important transcriptional factors that play fundamental roles in a broad range of biological processes, from development and metabolism to reproductive health [6]. Direct ligand binding induces a conformational change in the receptor, allowing it to recruit cofactors in regulating transcription [7,8]. The ligands for nuclear receptors include metabolites, vitamins, and hormones, as well as xenobiotics. Many nuclear receptors already have one or more ligands currently used as medicines, and nuclear receptors represent well-validated drug targets for several human diseases, including metabolic syndrome and hormone-dependent cancers (Table 1).

Two important concerns for drug development are efficacy and clinical safety, which are often associated with cross-activity of the compounds with undesired targets. Therefore, all lead compounds or drug candidates need to be assessed for toxicity to and selectivity for related targets. A major goal in nuclear receptor-targeting drug development has been to obtain ligands that exhibit regulatory activity in a receptor-selective manner with reduced adverse side effects. In this review, strategies to determine potential marine natural products as nuclear receptor modulators, the interaction between marine natural products and nuclear receptors, and potential marine natural products for drug development will be discussed and explored.

**Table 1:** Disease relevance and drug development of human nuclear receptors.

NR	Related Diseases	Drug Development
CAR	cholestatic liver disease [9]	Phenobarbital [12]
	type 2 diabetes [10]	
	hematopoietic malignancies [11]	
ER(α, β)	breast cancer [13] ovarian cancer, colon cancer [14] prostate cancer [15]	Bazedoxifene [16]
		Tamoxifen [17]
		Raloxifene [18]
		Lasofoxifene [19]
FXR	biliary cirrhosis, non-alcoholic fatty liver disease [9]	Fexaramine GW4064 [20]
		INT-747 [21]
GR	allergic, inflammatory, haematological disorders [22]	Dexamethasone [23]
		RU486 [24]
HNF4α	maturity onset diabetes of the young [25]	MEDICA 16 [26]
LXR(α, β)	non-alcoholic fatty liver disease [27]	GW3965 [31] *N*-Acylthiadiazolines [32] T00901317 [33]
	Alzheimer’s disease [28]	
	breast cancer [29]	
	atherosclerosis [30]	
PPAR(α, β, γ)	dyslipidemia [34] diabetes [35]	Fibrates [36]
		GW9662, GW501516 [37]
		Rosiglitazone [38]
		Thiazolidinediones [39]
PXR	endothelial detoxification [40]	Rifampicin [43]
	liver injury [41]	
	cholestatic liver disease [9]	
	cancers [42]	
RXR	metabolic diseases [44]	Bexarotene [46]
	cancers [45]	
TR(α, β)	thyroid hormone resistance syndrome [47]	Levothyroxine [49]
	thyroid cancer [48]	Liothyronine
VDR	diabetic nephropathy, hypertension, atherosclerosis [50,51,52]	Doxercalciferol [53]
MR	cardiovascular disease [54]	
	chronic kidney disease [55,56]	
	vascular Disease [57]	
PR	breast cancer [58,59]	RU-486 [24]
	endometriosis [60]	
AR	androgen insensitivity syndrome [61]	
	prostate cancer [62]	
	osteoporosis [63]	
RAR(α, β, γ)	acute promyelocytic leukemia [64]	
	kidney disease [65]	
	Alzheimer’s Disease [66]	
	skin diseases [67]	
	cancer [44]	

## 2. Nuclear Receptors: Structure and Function

**Figure 1:**
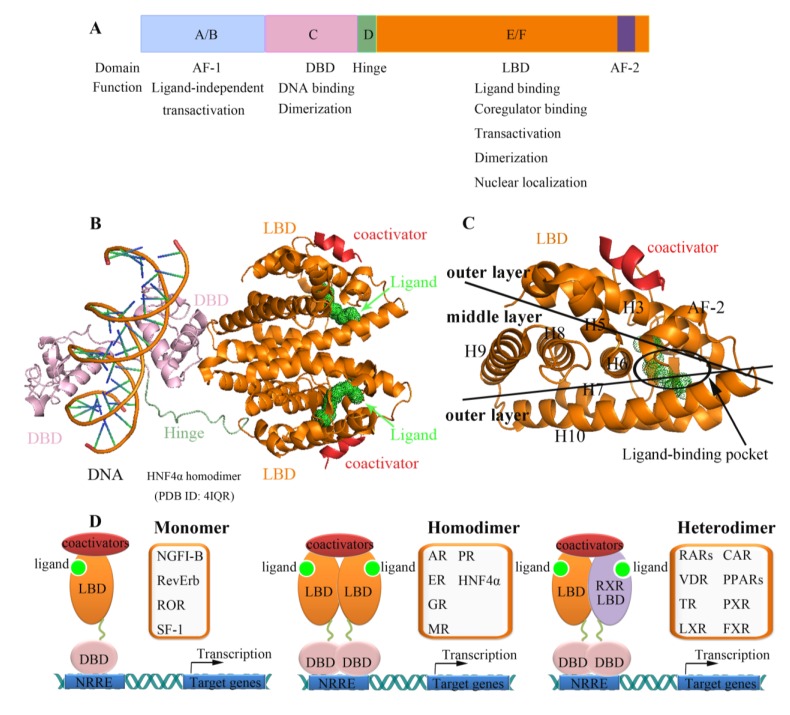
Structural and functional organization of nuclear receptors. (**A**) Schematic diagram for a common domain structure of NR. *N*-terminal A/B domain includes activation function 1 (AF-1), which mediates ligand-independent transcriptional activation. DNA binding domain (DBD) dictates specific response element recognition. Hinge region (Hinge) links DBD and LBD. *C*-terminal E/F domain encompasses the ligand-binding domain, which mediates ligand-dependent coactivator interactions; (**B**) Multi-domain structure of the HNF4α/DNA complex in cartoon representation. The crystal structure of HNF4α homodimer (PDB 4IQR) includes DBD (pink), Hinge (green), LBD (orange) in complex with response DNA sequence (left) and ligand (green dots); (**C**) Enlarged view of HNF4α LBD monomer, which clearly shows the three layer sandwich structure; (**D**) Metabolic regulation of NR. Ligand-activated NR complex recruits coactivator proteins that increase transcriptional activity of the gene. NRs bind DNA as monomers, homodimers or heterodimers.

Nuclear receptors can be divided into three groups: hormone receptors, adopted orphan receptors, and orphan receptors. They share high sequence identity and conserved domains. A typical nuclear receptor usually contains four functional regions: The A/B region (*N*-terminal activation function-1 domain, AF-1), the C region (DNA-binding domain, DBD), the D region (hinge region), and the E/F region (ligand-binding domain, LBD) (Figure 1A,B) [8]. Among these regions, the DBD and LBD are the most conserved. The LBD contains dimerization motifs and an activation function-2 (AF-2), located at the *C*-terminus of the receptor, in which conformation is highly dependent on ligand binding (Figure 1C). The LBD interacts with ligands and mediates transcriptional activation in a ligand-dependent fashion. Specifically, the binding of ligands to the LBD determines the recruitment of transcriptional coregulators that trigger the induction or repression of target genes (Figure 1D). As ligand binding and ligand-mediated cofactor recruitment are crucial for functions mediated by nuclear receptors, the LBD plays a critical role in nuclear receptor signaling. Thus, the LBD has been the focus of structural study, which has revealed important clues to the binding of ligands and cofactors [68,69,70,71].

**Figure 2:**
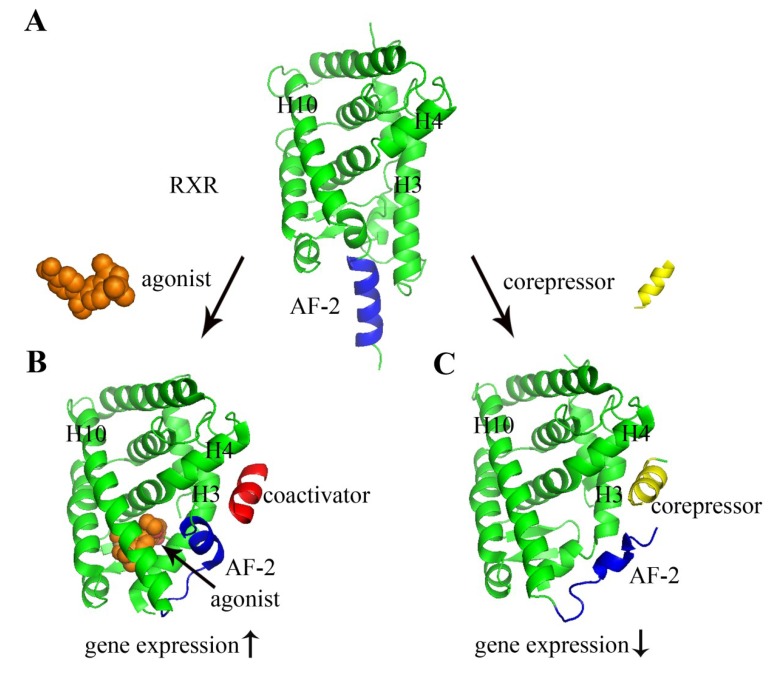
Structural basis of nuclear receptor ligand binding and cofactor recruitment. The structures shown here are the LBD of RXR (green image in the diagram). (**A**) Apo-RXR (no ligand bound, PDB 1LBD) [72]; (**B**) RXR complexed with agonist BMS649 (PDB 2ZY0) [73]; (**C**) RXR complexed with corepressor SMRT (silencing mediator for retinoid or thyroid-hormone receptors) (PDB 3R29) [74]. The agonist, coactivator, and corepressor are depicted as orange space filling spheres, a red image, and a yellow image, respectively. When an agonist is bound to a NR, the *C*-terminal α helix of the LBD (AF-2, blue) changes its position so that a coactivator protein (red) can bind to the surface of the LBD (**B**). Antagonist occupies the same ligand-binding cavity of the NR (antagonist not shown). However, antagonist ligands in addition have a side chain extension, which sterically pushes AF-2 to move towards outside, and corepressor (yellow) occupies roughly the same position in space as coactivators bind. Hence, coactivator binding to the LBD is blocked.

All nuclear receptors exhibit similar structural features (Figure 1B). Nuclear receptor LBD structures contain 11–13 α-helices that are arranged into a three-layer antiparallel α-helical sandwich [75,76]. The three long helices (H3, 7, and 10) form the two outer layers, and the middle layer of helices (H5, 6, 8, and 9) is present only in the top half of the domain, thereby creating a cavity for ligand binding, the so-called ligand-binding pocket (Figure 1C). The AF-2 also forms a helix that can adopt multiple conformations depending on different bound ligands (Figure 2). The first step of nuclear receptor activation is initiated by ligand binding, which induces a conformational change in the receptor; thus, the ligand-binding pocket is an important structural feature of nuclear receptors. Upon the binding of an agonist, nuclear receptors use a charge clamp pocket, in part composed of the *C*-terminal AF-2 helix, to form a hydrophobic groove for binding of the LXXLL motif of coactivators, such as SRCs (steroid receptor coactivators) and GRIP1 (glucocorticoid receptor interacting protein 1), leading to the modulation and promotion of gene transcription (Figure 1D). Antagonists block the effect of agonist through competitive binding to the same binding site in the nuclear receptor. Therefore, the antagonist-bound receptor is in an inactive state and preferentially binds corepressor proteins, leading to the repression of gene transcription [77,78]. The corepressors bind to LBDs via a conserved LXXXIXXXL/I motif, which is longer than LXXLL coactivator motif and adopts a three-turn α helix. The binding of corepressor motif induces major conformation change of AF-2 helix to accomadate the larger corepressor helix. The conformational flexibility of AF-2 helix allows the NR to sense the presence of the bound ligand, either an agonist or an antagonist, and to recruit the coactivator or corepressors that ultimately determine the transcriptional activation or repression of NRs (Figure 2) [8].

There is a pressing need to develop detailed structure–function relationships (SAR) of nuclear receptor and ligand interaction to facilitate the discovery of potent ligands. Structural comparison and analysis show that several features of the ligand-binding pocket have contributed to the ligand binding affinity and specificity. The ligand-binding pocket is the least conserved region on LBD, in which size and shape varies greatly from receptor subtype to subtype, to further accommodate specific ligands. The small pocket seen in the ERRα (estrogen-related receptor α) suggests that only ligands with four to five carbon atoms or less can fit [79]. In contrast, the large pocket in PXR (pregnane X receptor) allows the binding of antibiotic rifampicin, one of the largest structural ligands for nuclear receptors [80]. The overall hydrophobic nature of the ligand-binding pocket allows the NRs to interact with many lipid soluble ligands [81,82]. Given the plastic nature of the ligand-binding pockets, NRs respond differently to distinct ligands and readily exchange their ligands in different environments. From the drug discovery point of view, NRs may possess even greater potential as the flexible ligand-binding pocket allowing them to interact with a wider array of pharmacophores. As such, the ligand-binding pockets of nuclear receptors are promising sites for drug discovery research.

NR dimerization is critical in many regulatory processes, as NRs can bind to their cognate sequence-specific promoter elements on target genes either as monomers [83,84,85,86], homodimers [72,87,88,89,90,91,92,93], or heterodimers with RXRs (retinoid X receptor α, β, and γ) [75,94,95,96,97,98,99,100] (Figure 1D). Cooperative DNA binding and distinct recognition sites of homodimer and heterodimer make dimerization a general mechanism to increase binding site affinity, specificity, and diversity [101]. NR LBD stabilizes the dimers, while NR DBD contributes to response element selection by dictating the response element repertoire for monomer, homodimer, or heterodimer receptors. The steroid receptors appear to function as homodimers, such as ER (estrogen receptor) [88], PR (progesterone receptor) [102], AR (androgens receptor) [103], GR (glucocorticoids receptor) [93], and MR (mineralocorticoid receptor) [71]. HNF4α (Hepatocyte nuclear factor 4 alpha) is rather unique in that it binds DNA exclusively as a homodimer and, yet, behaves as the subtype nuclear receptors that localized primarily in the nucleus and usually activated as heterodimer with RXR [92]. One third of known NRs act as heterodimers with RXR, including RARs (retinoic acid receptors) [94,95], VDR (vitamin D receptors) [104,105], TR (thyroid hormone receptors) [96], LXR (liver X receptor) [97], CAR (constitutive androstane receptor) [98,99], PPARs (peroxisome proliferator activated receptors) [75,100], PXR, and FXR (farnesoid X receptor). Further, RXR self-associates into a homodimer or a homotetramer in the active or auto-repressed state [106]. It is suggested that RXR exists predominately in inactive homotetramer in the absence of ligand *in vivo* and dissociates upon ligand binding to form homodimer or heterodimers with other NRs [107]. Crystal structures of homodimers and heterodimers of NRs have revealed the structural organization of NR dimers. The NR dimerizations are mainly mediated by the dimerization surface located on the LBDs, which are topologically conserved. The dimeric arrangements are closely related, with residues from helices H7, H9, and H10, and loops L8–9 and L9–10 of each protomer, forming an interface comprising a network of complementary hydrophobic and charged residues [94]. NGFI-B (Nerve Growth factor IB) [84], RevErb [85], ROR (RAR-related orphan receptor) [83], SF-1 (steroidogenic factor 1) [86], and several other orphan NRs have been shown to bind DNA as monomers. Interestingly, some NRs have been reported to function in multiple patterns. For example, TR can bind to DNA as monomers, homodimers, or heterodimers. A single surface mutation, D355R, was shown to be crucial for converting the modestly stable monomeric TR LBD into a stable dimer [108]. LXR have been reported both as homodimers and heterodimers, and the comparison of these two different dimer patterns explains differences in dimer affinity and leads us to propose a model for allosteric activation in LXR dimers, in which an unactivated RXR partner provides an inhibitory tail wrap to the cofactor binding pocket of LXR [109]. When activated, ER translocates into the nucleus, binding to DNA either as a αα homodimer or as a αβ heterodimer [110,111].

## 3. Nuclear Receptors as Drug Targets in Related Disease Signaling

Extensive studies have revealed that nuclear receptors are involved in many metabolic and inflammatory diseases, such as diabetes, dyslipidemia, cirrhosis, and fibrosis [112,113,114,115,116,117]. As ligands play a pivotal role in modulating nuclear receptor activity, agonists or antagonists of nuclear receptors have been suggested for pharmaceutical development. The examples of disease relevance of NRs and drug development are listed in Table 1. As most marine natural ligands have been reported to target PPARs, FXR, PXR, and RARs, the following discussion focuses on the drug discovery targeting these well-described NRs as well as their therapeutic uses.

### 3.1. Peroxisome Proliferator-Activated Receptor (PPAR)

Peroxisome proliferator-activated receptors (PPARs, isoforms α, β/δ, and γ) are ligand-activated nuclear receptors that play essential roles in lipid homeostasis [34], adipocyte differentiation [118], and insulin responses [119]. A large ligand-binding pocket is a distinguishing feature of PPARs, which allows them to bind a variety of chemical ligands including fatty acids, fibrates, and the thiazolidinedione class of antidiabetic drugs with diverse shapes, sizes, and compositions. The binding of ligands causes a conformational change in PPARs and the recruitment of coregulators, such as PGC1α (peroxisome proliferator-activated receptor gamma coactivator 1-alpha), which results in the transcriptional regulation of downstream target genes [120,121,122,123]. These genes in turn regulate many metabolic pathways involved in glucose homeostasis and insulin sensitivity. PPARα is expressed in the liver, heart, muscle, and kidneys, and it regulates fatty acid metabolism and transport. PPARγ is expressed in adipose, muscle, and macrophage and is critical for adipogenesis and lipid storage. PPARδ is broadly expressed in the body and is involved in fat oxidation, energy expenditure, and lipid storage. These biological roles have made PPARs important targets in the treatment of metabolic syndrome and diabetes.

The most extensively studied ligands for PPARs are thiazolidinediones (TZDs), a class of drugs used to increase insulin sensitivity. TZDs can decrease insulin resistance, modify adipocyte differentiation, and induce lipoprotein lipase (LPL) by regulating the expression of PPARγ target genes [124,125,126,127]. However, TZDs are clinically limited due to severe adverse effects, such as fluid retention, weight gain, liver toxicity, and cardiovascular disease [38,128,129]. Therefore, it is imperative to develop improved PPARs ligands that retain the benefits in improving insulin resistance but that have reduced side effects. Current approaches include multi-target strategies (ligands targeting more than one PPAR isoform) and selectivity strategies (selective PPARγ modulators (SPPARMs)). Some SPPARMs with partial or no agonism in transcriptional activity have shown similar glucose-lowering effects to rosiglitazone but with reduced side effects [69,130,131,132]. Recently, we reported two novel ligands for PPARs (RU486 and ionomycin) as partial agonists for PPARγ [69,133], which may provide promising therapeutic agents targeting PPARs.

### 3.2. Farnesoid X Receptor (FXR)

Farnesoid X receptor (FXR), also known as bile acid receptor, is important in maintaining bile acid and cholesterol homeostasis. FXR regulates the expression of transporters and biosynthetic enzymes, such as cholesterol 7α-hydroxylase (CYP7A1), which is crucial for the physiological maintenance of bile acid homeostasis [134,135,136]. FXR is highly expressed in the liver, intestine, kidneys, and adrenal gland [137,138,139] and is activated by chenodeoxycholic acid (CDCA) and other bile acids [140,141]. Following ligand binding, the transcriptional function of FXR is mediated through the recruitment of coactivators such as SRC1 or through the release of specific corepressors such as NCoR1 (nuclear receptor corepressor 1) and SMRT [142,143,144]. FXR regulates lipid metabolism, possibly by interacting with PPARα and PPARγ, as well as repressing sterol regulatory element-binding protein-1c (SREBP-1c) [145,146,147]. Activation of FXR by an agonist or hepatic overexpression of FXR lowered blood glucose levels in both diabetic db/db and high-fat diet-fed wild-type mice, and FXR-null mice exhibited glucose intolerance and insulin insensitivity [148].

Given the important roles of FXR in physiological and pathological processes, FXR ligands have become promising therapeutic agents for different diseases. Synthetic agonists of FXR (including GW4064, INT-747, and fexaramine) have been developed to treat primary biliary cirrhosis and non-alcoholic fatty liver disease [149]. However, synthetic FXR ligands have limitations owing to side effects and uncertain bioavailability. The application of known natural FXR ligands, such as bile acid CDCA, is also limited by their poor selectivity and low affinity [144].

### 3.3. Retinoic Acid Receptor (RAR)

Retinoic acid receptors (RARs) exist as three subtype isoforms (α, β, and γ) that collectively contribute to a response to both natural and synthetic ligands [150,151]. RARα is associated with differentiation therapy for human acute promyelocytic leukemia (APL) [44,152]. RARβ plays a crucial role in limiting the growth of different tumor cell types and is thus a promising target for the treatment of breast and other cancers [153]. RARγ is primarily expressed in the skin and is involved in skin diseases, such as psoriasis and acne [67].

RARs activate transcription in a ligand-dependent manner by binding to DNA as heterodimers with RXR. Ligand activation of RAR/RXR heterodimers drives physical interactions with co-regulatory proteins (corepressors and coactivators) and binding to retinoic acid response elements (RAREs) present in the promoter or enhancer regions of target genes. RAR ligand retinoids, which include vitamin A and its derivatives, have demonstrated some success as therapeutic agents for a wide range of diseases [150,151,154,155,156,157,158]. Retinoids exert their therapeutic effect by activating retinoid receptors, including RARs (α, β, and γ) and RXRs (α, β, and γ) [159,160,161]. For instance, the use of all-trans retinoic acid (ATRA), a retinoid panspecific for all RARs, has been very successful in the treatment of APL by inducing differentiation of leukemic cells.

Due to their teratogenic properties, retinoids can result in a number of undesired side effects, such as increased serum triglycerides and bone toxicity, presumably due to their panspecific activation of all RAR isoforms. Further, the occurrence of RA resistance in a variety of cancer cells is also one of the major concerns with retinoid treatments, which hampers RA-based chemotherapy [33]. Consequently, there is an urgent need to develop ligands against RARs distinct from retinoids, which may yield more efficacious RAR-targeted drugs with less adverse effects.

### 3.4. Pregnane X Receptor (PXR)

By sensing the presence of foreign toxic substances, PXR can up-regulate the expression of proteins involved in the detoxification and clearance of these substances from the body [162]. In addition to detoxification and metabolism of xenobiotics, PXR is also involved in various physiological and pathophysiological processes, such as lipid metabolism [163], glucose homeostasis, and inflammatory response [164]. Recent studies suggest that PXR may be a useful target for pharmacological therapies in various conditions, including liver disease [165], and inflammatory bowel diseases (IBD), encompassing Crohn’s disease (CD) and ulcerative colitis (UC) [164,166]. PXR is activated by a large number of endogenous and exogenous chemicals including steroids, antibiotics, antimycotics, bile acids, and many other herbal compounds [162]. Following ligand binding, PXR forms a heterodimer with RXR and binds to specific PXR response elements (PXREs), located in the *N*-terminal flanking regions of PXR target genes, resulting in their transcriptional activation [167]. Primary targets of PXR activation are P450 enzymes (CYP3A, CYP2C, and CYP2B), important phase I oxidative enzymes that are responsible for the metabolism of many drugs [167,168]. In addition, PXR up-regulates the expression of phase II conjugating enzymes that improve solubility of phase I metabolites (glutathione S-transferases [169], sulfotransferases, and UDP-glucoronosyltransferases [170,171]) and phase III transport uptake and efflux proteins, such as OATP2 [172] and MDR1 [173,174].

PXR LBD shows a typical NRs organization, but its ligand-binding pocket is substantially larger than those of many other NRs [175,176]. Therefore, PXR is able to bind both small and large ligands. The number of chemicals that are reported to activate PXR has grown rapidly, including many drugs currently in use, such as statins, the antibiotic rifampicin, antihypertensive drugs nifedipine and spironolactone, anticancer compounds, HIV protease inhibitors, calcium channel modulators, diverse environmental toxicants, such as plasticizers and pesticides [177]. Rifampicin, a semisynthetic PXR agonist, is currently used in the treatment of cholestatic liver disease and its exact mechanism of action is still under investigation. Notably, most PXR ligands reported show agonism properties, whereas to date only few PXR antagonists have been identified [178].

## 4. Strategies for the Discovery of Novel Ligands for Nuclear Receptors

The discovery of novel ligands for nuclear receptors represents an interesting and promising therapeutic approach to various diseases. Both indirect and direct methodologies have been developed to identify compounds that bind to nuclear receptors *in vitro*, generally involving the LBDs. Direct approaches are the use of high-throughput screening (HTS) assays to identify compounds capable of regulating nuclear receptors; these assays have become increasingly popular because they can rapidly and accurately distinguish compounds among large chemical libraries [179]. Improved methods for the synthesis of chemical libraries have created a need for increased sensitivity and throughput in screening [180,181]. In the field of HTS, there are often various biochemical assays and cell-based assays available for efficiently measuring a particular nuclear receptor-ligand interaction; such choices are fluorescence polarization (FP), AlphaScreen assays, and transactivation reporter gene assays.

### 4.1. Fluorescence Polarization (FP) and Fluorescence Resonance Energy Transfer (FRET)

Fluorescence polarization (FP) is one of the standard ligand-binding assays, and it is commonly deployed in a high-throughput format to measure the rotational speed of a fluorophore during its fluorescence lifetime, defined as the duration of time post-excitation by plane-polarized light but prior to light emission [182]. In an FP assay, compounds are screened for their ability to compete with a labeled, validated LBD-binding ligand. Either an agonist or antagonist can be detected in this type of competitive binding assay [183]. A small coactivator peptide alone, containing an LXXLL motif and a fluorophore label, rotates quickly, and exhibits low polarization. Ligand binding of nuclear receptors induces the formation of a ligand-nuclear receptor-coactivator complex, which is larger and rotates more slowly, resulting in the emission of more highly polarized light. Therefore, ligand binding can be quantitatively monitored based on the difference in polarization.

The FRET assay was developed from the FP assay, in which the fluorescent signal intensity depends upon the interaction between a fluorescently labeled LBD and coactivator proteins. A fluorescent signal is obtained when the LBD is in close proximity to the coactivator proteins through its interaction with a putative agonist. In a time-resolved FRET (TR-FRET) assay, lanthanide chelates are used as the donor fluorophore and may be used to label either the protein directly or an antibody to a common protein tag. A receptor ligand is labeled with fluorescein or some other suitable acceptor fluorophore. Potential ligands that compete for LBD binding will result in an associated decrease in the TR-FRET signal. TR-FRET assays can greatly reduce data variability because they are able to measure both lanthanide and acceptor fluorophore emissions to generate FRET ratios. The FRET ratio is disrupted when a competitor ligand binds to the LBD and displaces the bound fluorescein-labeled tracer molecule. TR-FRET assays can minimize the nonspecific interference derived from short fluorescent lifetime components such as plate plastics, compound autofluorescence, and diffusion-enhanced FRET. Moreover, selecting the proper donor and acceptor fluorophores and wavelength filters allows for the monitoring of two simultaneous processes [184]. However, FP assays also have several disadvantages, including their high level of background, which translates into a lower signal-to-noise ratio and decreased sensitivity, and their inability to distinguish between agonists and antagonists [185].

### 4.2. AlphaScreen (Cofactor Binding Assays)

AlphaScreen, used mostly in high throughput screening, is a homogenous assay technology similar to TR-FRET. AlphaScreen technology was first described in 1994 and is based on the principle of luminescent oxygen channeling [186,187]. AlphaScreen is a bead-based, nonradioactive amplified luminescent proximity homogeneous assay in which a donor and an acceptor pair of 250-nm-diameter reagent-coated polystyrene microbeads are brought into proximity by a molecular interaction of binding partners immobilized to these beads [187,188,189]. The detection system of AlphaScreen can be time-gated, as the signal is long lived, thus, eliminating short-lived background signals. The high sensitivity of the assay derives from the very low background fluorescence. Furthermore, the detection wavelength is shorter than the excitation wavelength, thereby further reducing the potential for fluorescence interference.

The larger diffusion distance of the singlet oxygen makes the available detection of binding distance 200 nm, whereas TR-FRET is limited to 9 nm [190]. The most important advantage of AlphaScreen over TR-FRET is that the AlphaScreen can distinguish between an agonist and an antagonist by the selective usage of coactivator or corepressor peptides. The AlphaScreen system is generally applicable over a wide variety of biomolecular targets, which can supplant solid-support binding assays in many applications, such as receptor-ligand interactions [191], lipid signaling [192], protein kinase monitoring [193], and other types of signaling [194].

The unique advantages of AlphaScreen have made it an excellent alternative to TR-FRET for the measurement of ligand-induced nuclear receptor-cofactor interactions [195]. A comparison study between AlphaScreen and TR-FRET suggested that AlphaScreen would be better because of its increased sensitivity, decreased plate reading time, and increased proximity limits [195]. The large signal/background ratio and increased sensitivity in the AlphaScreen assay enable a significant reduction in the quantities of nuclear receptor protein and biotinyl-cofactor required for screening. For the AlphaScreen format, acceptable data can be obtained with five-fold less of these reagents compared to the TR-FRET assays. In recent years, more and more NR ligands have been identified by AlphaScreen. Our lab also uses AlphaScreen to determine the binding of the various cofactor peptide motifs to nuclear receptor LBDs in response to ligands. By using a hexahistidine detection kit from PerkinElmer (including the *N*-terminal biotinyl peptides, His-tag fusion LBD, and compound libraries) [68,69,133,196,197,198], several ligands for various nuclear receptors have been identified, including two PPARγ agonists [69,133], a marine natural product as an RAR agonist [196], an existing drug as an FXR agonist [68], a dual PPARα and PPARδ agonist [198], and a natural compound as an agonist for orphan receptor RORγ [197].

### 4.3. Transactivation Reporter Gene Assays (Transient Transfection Assays)

Cell-based systems are also widely used for identifying ligands that interact with nuclear receptors. Transient and stable transfections are two types of cell-based systems for assessing nuclear receptor transactivation. The most common method for evaluating nuclear receptor activation is transient reporter gene assay, through transient transfection of a nuclear receptor together with a cognate response element-reporter gene construct. In Gal4-driven reporter assays, the cells are transfected with Gal4-LBDs of various nuclear receptors and pG5Luc reporter. In native promoter reporter assays, the cells are co-transfected with plasmids encoding full-length nuclear receptors and their cognate luciferase reporters (e.g., PPARs and PPRE). Many cell lines are available to serve as recipients of these plasmids, including COS7, HuH7, HEK293, HepG2, and other stable tumor cell lines. Many nuclear receptor agonists have been identified using transient transactivation systems [199,200,201]. The advantages of reporter gene assays are their ease of use, efficiency, and reproducibility, as well as their ability to differentiate mechanisms of action in the nuclear receptor application. Similar to biochemical studies, the cell based transactivation assays can also be employed to obtain EC_50_ values that reflect the potency of a compound. This is important as clinical models are based upon EC_50_ and *E_max_* values to rank a compound’s potency [202].

The biochemical assays, including FP, FRET, and AlphaScreen assays are straightforward, fast and easy to set up, with reasonable cost, enabling the high-throughput screening of a large number of chemical libraries. Following biochemical screening, it’s also necessary to perform cell-based assays, like a transactivation reporter assay, to validate the functional relevance of the hit compounds. To further characterize the underlying molecular mechanisms, various other biological assays and structure-activity relationships (SAR) analysis are often critical to gather more insights to optimize the hit compounds for future drug discovery. For example, Chromatin Immunoprecipitation (ChIP) is a type of immunoprecipitation experimental technique used to investigate coactivators and corepressors recruitment modulated by ligands [200,203,204], while real-time PCR allows precise quantification for the expression pattern of nuclear receptor target genes regulated by the hit compounds [68,69,200,204].

## 5. Search for Marine Natural Products Targeting Nuclear Receptors

The value of natural products from marine species has been recognized for over half a century, but it is only in recent years there has been a renewed interest in this potential source of new medicines [205]. Chemical, structural, and pharmacological characterizations of marine nature products libraries have successfully identified many hit compounds that regulate NRs. Recent research has been focusing on the development of novel drugs specifically targeting nuclear receptors for treating a variety of diseases, such as cancer, diabetes, dyslipidemia, fatty liver disease, drug hepatotoxicity, and cholestasis. Searching for novel nuclear receptor ligands (agonists and antagonists) from marine natural products with improved selectivity will prompt the exploration of the extraordinary chemical diversity associated with natural products. The marine environment has provided a rich source of nuclear receptor ligands, and a number of natural products have been shown to display remarkable affinity for nuclear receptors, in some cases with unique modes of action (Table 2). These nuclear receptors proven to be targets of marine natural products include RAR [196], FXR [206], PPARs [207,208], AR [209], GR [210], VDR [211], PR [212], and PXR [213]. In this section, selected examples of the marine natural ligands for NRs will be described with an emphasis on their therapeutic potentials.

**Table 2:** List of marine natural molecules targeting nuclear receptors signaling.

Compounds	Origin	Target(s)	Comments/References	Method
luffariellolide	Marine sponges *Luffariella* sp*.* and *Fascaplysinopsis*	RAR	agonist of RAR with inhibitory effects on cancer cells [196]	AlphaScreen
7-hydroxy retinoic acid	cyanobacteria *Microcystis aeruginosa* and *Spirulina* sp.	RAR	agonist of RAR [214]	yeast two hybrid
SQA	Brown alga *Sargassum yezoense*	PPARα/γ	PPARα/γ dual agonists [207]	transfection assay
SHQA	Brown alga *Sargassum yezoense*	PPARα/γ	PPARα/γ dual agonists [207]	transfection assay
Ionomycin	*Streptomyces conglobatus*	PPARγ	partial agonist of PPARγ [69]	AlphaScreen
Tuberatolide A	Korean marine tunicate *Botryllus tuberatus*	FXR	antagonized the (CDCA)-activated FXR [206]	transfection assay
Meroterpenoids tuberatolide B	Korean marine tunicate *Botryllus tuberatus*	FXR	antagonized the (CDCA)-activated FXR [206]	transfection assay
2′-epi-tuberatolide B	Korean marine tunicate *Botryllus tuberatus*	FXR	antagonized the (CDCA)-activated FXR [206]	transfection assay
yezoquinolide	Korean marine tunicate *Botryllus tuberatus*	FXR	antagonized the (CDCA)-activated FXR [206]	transfection assay
(*R*)-sargachromenol	Korean marine tunicate *Botryllus tuberatus*	FXR	antagonized the (CDCA)-activated FXR [206]	transfection assay
(*S*)-sargachromenol	Korean marine tunicate *Botryllus tuberatus*	FXR	antagonized the (CDCA)-activated FXR [206]	transfection assay
Compounds 1–5	marine sponge *Spongia* sp.	FXR	FXR antagonistic activity [215]	transfection assay
4-methylenesterols	marine sponge *Theonellaswinhoei*	FXR, PXR	potent agonists of PXR and antagonists of FXR [213,216]	transfection assay
Conicasterol E	marine sponge *Theonella swinhoei*	FXR, PXR	dual FXR and PXR agonist [217]	transfection assay
Malaitasterol A	marine sponge *Theonella swinhoei*	PXR	potent agonists of PXR [218]	transfection assay
suvanine	marine sponge	FXR	antagonist of FXR [200]	transfection assay
sulfated sterol (compound 8)	marine invertebrates	FXR	antagonist of FXR [219]	transfection assay
solomonsterols A and B	marine sponge *Theonellaswinhoei*	PXR	agonist of PXR [220]	transfection assay
okadaic acid	microalgae	CiVDR/PXRa, hPXR	activation at nanomolar concentration [211]	transfection assay
pectenotoxin-2	microalgae	CiVDR/PXRa	activation at nanomolar concentration [211]	transfection assay
Phosphoiodyns A	Korean marine sponge *Placospongia* sp.	PPARδ	highly potent hPPARδ activity (EC_50_ = 23.7 nm) [208]	NMR spectrum
Herdmanine I and K	marine ascidian *Herdmania momus*	PPARγ	similar PPARγ agonistic activities to rosiglitazone [221]	transfection assay
gracilioether B and plakilactone C	marine sponge *Plakinastrella mamillaris*	PPARγ	selective PPARγ ligands [222]	transfection assay
Niphatenones	Marine sponge *Niphates digitalis*	AR	block androgen receptor transcriptional activity in prostate cancer cells [209]	transfection assay
Psammaplin A	marine sponge *Pseudoceratina rhax*	PPARγ	activates PPARγ in a MCF-7 cell-based reporter assay [223]	transfection assay
chlorinated peptides sintokamides A to E	sponge *Dysidea* sp.	AR	inhibitor of *N*-terminus transactivation of the androgen receptor in prostate cancer cells [224]	transfection assay
theonellasterol	marine sponge *Theonella swinhoei*	FXR	FXR antagonist [225]	transfection assay
steroids 3-oxocholest-1,22-dien-12beta-ol and 3-oxocholest-1,4-dien-20beta-ol	soft coral *Dendronephthya gigantea*	FXR	inhibitory activity against FXR with IC(50)’s 14 and 15 µM [226]	transfection assay
Bendigoles D	marine sponge derived bacterium *Actinomadura* sp. *SBMs009*	GR	inhibitor of GR [210]	transfection assay
(3*R*)-cyclocymopol monomethyl ether	marine alga *Cymopolia barbata*	PR	PR antagonist [212]	transfection assay
(3*S*)-cyclocymopol monomethyl ether	marine alga *Cymopolia barbata*	PR	PR agonist [212]	transfection assay

### 5.1. Luffariellolide and RARs

Very few reports on natural ligands of RARs have been reported so far in the literature [196,214,227]. Among these natural ligands, 7-hydroxy retinoic acid and luffariellolide were isolated from marine organisms. 7-hydroxy retinoic acid is an analog of ATRA isolated from cyanobacteria *Microcystis aeruginosa* and *Spirulina* sp., and its relative RAR agonistic activity was lower than ATRA [214]. Due to its similar structure with RA and RA resistance in a variety of cancer cells, further exploration of 7-hydroxy retinoic acid in therapy has been limited [228]. Therefore, the search for new RAR ligands other than retinoids with distinct activity profiles and fewer side effects may provide a new rational drug design strategy targeting nuclear receptor RARs. Meeting these criteria, luffariellolide may be an ideal hit compound for drug design [196].

Considering the structural diversity, lower toxicity, and abundance of marine natural products, our laboratory set out to search for novel ligands for RARs present in marine products libraries by using the AlphaScreen biochemical assay [196]. The marine natural product luffariellolide, a hexane extract isolated from sponges of *Luffariella* sp. and *Fascaplysinopsis*, was identified as a positive RARα activator. Notably, the chemical structure of luffariellolide shows a unique γ-hydroxybutenolide ring terminus instead of a carboxylic acid moiety for retinoids, thus, representing a novel approach for an RAR ligand design strategy distinct from the retinoid scaffold. In the follow-up study, cell-based bioassays were used to attain characteristics of luffariellolide in activating nuclear receptors, and the results have shown that luffariellolide was a selective agonist for all three RARs, but not for other NRs, including the heterodimer partner RXRα.

**Figure 3:**
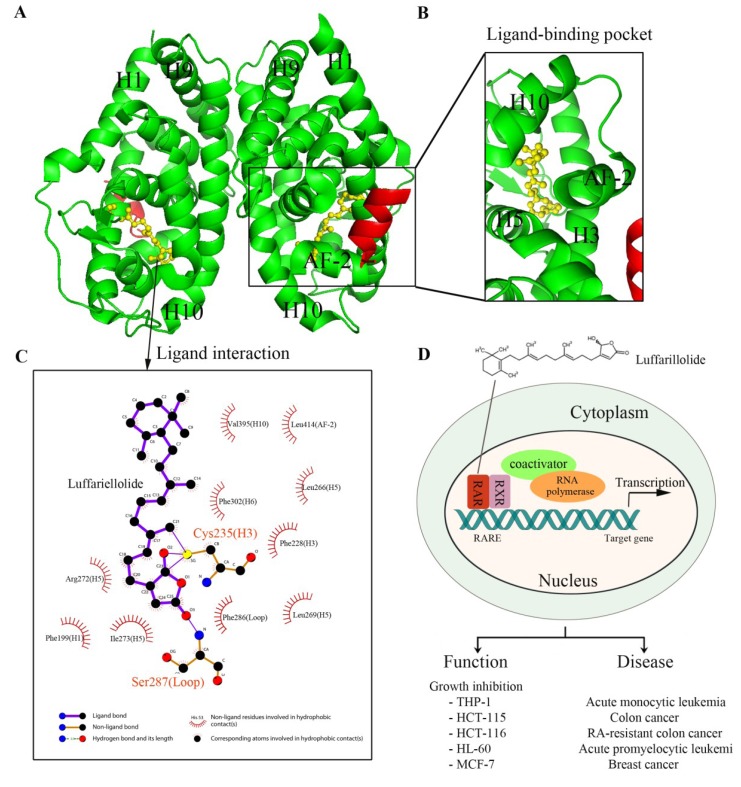
The structure and gene regulation of luffariellolide bound RARα. (**A**) Intact structure of RARα LBD-Luffariellolide complex. Luffariellolide-bound RARα adopts a dimer fold. The RARα LBD (green) and coactivator SRC1 (red) motif are depicted in image representation, and luffariellolide is shown in yellow ball and stick representation; (**B**) Enlarged view of the ligand-binding pocket of RARα. AF-2, together with Helix 3, Helix 5, and Helix 10, form a ligand-binding pocket for luffariellolide; (**C**) Interaction analysis of luffariellolide by Ligplot [229]. Cys^235^ from Helix 3 of RARα LBD forms a covalent bond with the ketone group of the γ-hydroxybutenolide ring terminus from luffariellolide; (**D**) Gene regulation by RARα in the presence of luffariellolide. Luffariellolide treatment can inhibit the cell proliferation of monocytic leukemia cell line THP-1, RA-sensitive colon cancer cell line HCT-115, RA-resistant colon cancer cell line HCT-116, promyeloid leukemic cell line HL-60, and breast carcinoma line MCF-7 [196].

The ability of luffariellolide to promote recruitment of coactivator motifs by RARs was determined and the results were consistent with that of the cell reporter assay. Moreover, we were also able to obtain the crystal structure of luffariellolide bound to the LBD of RARα, which revealed the molecular basis for the binding of luffariellolide by RARs (Figure 3A,B). By structural comparison between the luffariellolide-RARα complex and the ATRA-RARα complex, a unique binding mode of luffariellolide to RARα was identified. Strikingly, the luffariellolide-RARα structure revealed a covalent interaction between the ligand and the receptor, in addition to several hydrophobic and van der Waals interactions. Specifically, a covalent bond formed between the Cys^235^ in RARα and the ketone on the unique γ-hydroxybutenolide group of luffariellolide (Figure 3C). The covalent interaction has been further confirmed by mass spectrometry (MS) and by mutagenesis studies of RARα LBD. Incubation of RARα LBD with luffariellolide yielded a mass addition corresponding to the molecular mass of the luffariellolide ligand. In the mutagenesis study, both the C235A and C235L mutations abolished the activation of RARα by luffariellolide, suggesting the critical roles of this covalent modification for the receptor-luffariellolide interaction.

Next, several cell-based experiments were conducted to assess the roles of luffariellolide in regulating the physiological functions of RARs. Luffariellolide could reduce cell proliferation and induce known RA-inducible genes in various cancer cells (Figure 3D). Of significance is the observation that in an RA-resistant HCT-116 cell line, in which retinoids failed to show effect, luffariellolide was able to function as an RAR agonist, reducing cell proliferation and switching on target genes. In future studies, it will be worth investigating if luffariellolide can act in suitable *in vivo* disease models with the downstream target gene responsible for overcoming RA-resistance using luffariellolide as a probe.

Taken together, as a novel RAR agonist, marine natural product luffariellolide may provide an alternative drug design strategy for non-retinoid compounds with advantages over current RA drugs. The unique characteristics of the γ-hydroxybutenolide ring may represent a new pharmacophore that can be optimized for selectively targeting RARs.

### 5.2. Marine Products Targeting FXR

Marine sponges *Theonella* species have been proven to be an extraordinary source of unusual new chemical entities, mainly peptides and macrolides, with impressive biological activities [217]. Apart from anti-inflammatory peptides [230] and cytotoxic macrolides [231], recent chemical and pharmacological analysis of several *Theonella* extracts has furnished a large family of molecules able to target FXR and PXR, including solomonsterols [220,232] and a large number of 4-methylenesteroids [213,216,217,218]. Solomonsterols A and B were identified to stimulate the expression of CYP3A4 and MDR1, two well characterized PXR responsive genes, making them potential hit compounds for the treatment of human disorders characterized by inflammation and dysregulation of innate immunity [220,232]. Further pharmacological studies in animal models of colitis demonstrated that synthetic solomonsterol A effectively protects against development of clinical signs and symptoms of colitis and reduces the generation of TNFα, a signature cytokine for this disorder [232]. Within the family of 4-methylenesteroids, theonellasterol, the major component of the steroidal fraction of *Theonella swinhoei*, was identified as a highly selective FXR antagonist with pharmacological potential in the treatment of cholestasis [204]. Theonellasterol directly inhibits FXR transactivation caused by CDCA and reverses the effect of CDCA on the expression of canonical FXR target genes. In rodent models of cholestasis, theonellasterol attenuates liver injury caused by bile duct ligation. Interestingly, 4-methylenesterols derived from marine sponge *Theonella swinhoei*, was found to have potent PXR agonist activity and FXR antagonist activity [213,216,217,218]. The dual behaviors of these marine natural compounds may lead to combination therapies involving lower drug doses and therefore reduced side effects.

Hepatic FXR activation leads to both beneficial actions and potentially undesirable side effects such as the inhibitions of bile acids synthesis and basolateral efflux of bile acids [233]. These findings have raised the notion that FXR antagonists might be useful in the treatment of liver disorders caused by impairment of bile secretion [204]. However, only few FXR antagonists are known and the main contribution is derived from terrestrial and marine natural compounds [228]. Most of the FXR antagonists have a steroid skeleton [219,220,232,234,235]. The steroidal FXR antagonists may also regulate steroid receptors and are unsuitable for studying FXR physiology. Therefore, the discovery of nonsteroidal FXR antagonists is highly desirable.

Six nonsteroidal FXR antagonists from the Korean marine tunicate *Botryllus tuberatus* were identified, including the isoprenoid tuberatolide A, a pair of diastereomeric meroterpenoids (tuberatolide B and 2′-epi-tuberatolide B), and three meroterpenoids (yezoquinolid, (*R*)-sargachromenol, and (*S*)-sargachromenol) [206]. They show potent inhibition of FXR transactivation without significant cytotoxicity. More importantly, these compounds have no effects on steroid receptors in transactivation experiments. Structurally, the six compounds all have γ-lactones or carboxylic acids at the C-15 position, suggesting that the carbonyl group at C-15 may enhance the FXR antagonistic effects and may help to unravel the controversial function of FXR in atherosclerosis [206].

Sesterterpenes isolated from marine sponges are particularly interesting from their pharmacological properties. There is no report of biological activity of scalaranes in regard to metabolic disorders until the discovery of five novel scalarane sesterterpenes with FXR inhibitory activities [215]. All five sesterterpenes were isolated from marine sponge *Spongia* sp. and showed inhibitory activities against FXR transactivation. Notably, 12,24-diacetoxy-deoxoscalarin showed the most potent inhibitory activity with an IC_50_ value of 8.1 µM without any significant cytotoxicity [215]. In addition, suvanine, a furano sesterterpene sulfate from the marine sponge *Coscinoderma mathewsi*, was reported as a novel antagonist of the mammalian bile acid sensor FXR [200].

### 5.3. Marine Products Targeting PPARs and AR

As for PPARs, a master regulator of adipocyte differentiation, psammaplin A from the sponge *Pseudoceratina rhax* and herdmanine from the marine ascidian *Herdmania momus* were both revealed to activate PPARγ [223]. In addition, the marine natural products sargaquinoic acid (SQA) and sargahydroquinoic acid (SHQA) from *Sargassum yezoense* were reported as novel PPARα/γ dual agonists. SQA and SHQA increased adipocyte differentiation accompanied by increased expression of adipogenic marker genes, suggesting that these PPARα/γ dual agonists may reduce insulin resistance through regulating adipogenesis [207]. SQA, also named aleglitazar, already entered phase III clinical trials for the treatment of type 2 diabetes but failed due to its inacceptable side effects related to bone fractures, heart failure, and gastrointestinal bleeding [236]. Gracilioether B and plakilactone C isolated from the marine sponge *Plakinastrella mamillaris* were identified as selective PPARγ ligands in transactivation assays. Both agents regulate the expression of PPARγ-dependent genes in the liver and inhibit the generation of inflammatory mediators by macrophages. More importantly, these two marine natural compounds covalently bind to the PPARγ LBD through a Michael addition reaction involving a cysteine residue and the α,β-unsaturated ketone in their side chains, suggesting the possibility to develop novel PPARγ modulators as potential agents in the treatment of inflammatory disorders [222].

Interestingly, several marine natural products with antagonism activities interact with AR at its *N*-terminal domain, not the LBD, including a group of glycerol ethers (niphatenones) from the sponge *Niphates digitalis* [209] and a group of chlorinated peptides, termed sintokamides, from the sponge *Dysidea* sp. [224]. These two AR antagonists demonstrate the possibility of targeting regions other than the traditional LBDs to modulate NR activity, thus, providing an alternative method for NR ligand screening to overcome hormone resistance.

## 6. Conclusions

As the pathogenesis of diseases is complicated, the development of safe and effective drugs against diseases is full of challenges. Currently, nuclear receptors have been engaged in hit compound discovery as important targets involved in disease. In comparison with synthetic compounds, natural products possess multiple advantages for their large-scale structures and target diversity both in single target and signaling pathway-based drug discovery strategies. In addition, the complex structures of natural products lead to great target diversity. Therefore, natural molecules often function as good probe candidates for exploring novel targets or pathways involved in diseases.

The marine environment has long been known to be species-rich. Recent studies have revealed more and more marine natural products as nuclear receptor modulators, which highlights the translational possibilities of natural products in drug discovery. Many emerging strategies have been developed to speed up the drug discovery process, including natural product isolation technologies, compound synthesis and optimization methods, and high-throughput screening technologies. It is reasonable to expect that interaction between marine natural products and nuclear receptors will continue to provide more hit compounds, as well as a mechanistic understanding for drug discovery targeting nuclear receptors, thereby, greatly facilitating the development of therapeutic reagents against human diseases.

Taken together, the discovery of NR ligands in marine natural products and their derivatives opens a promising approach for the design and preparation of new potential leads in the pharmacological treatment of NR-mediated human diseases.
